# Facile Strategy for Synthesizing Non-Stoichiometric Monoclinic Structured Tungsten Trioxide (WO_3−*x*_) with Plasma Resonance Absorption and Enhanced Photocatalytic Activity

**DOI:** 10.3390/nano8070553

**Published:** 2018-07-21

**Authors:** Shihao Chen, Yang Xiao, Wei Xie, Yinhai Wang, Zhengfa Hu, Wei Zhang, Hui Zhao

**Affiliations:** 1 School of physics & optoelectronic engineering, Guangdong University of Technology, Guangdong 510006, China; shchenbhx@163.com (S.C.); xiaoyxy1023@163.com (Y.X.); weizh55@gdut.edu.cn (W.Z.); kkhui@gdut.edu.cn (H.Z.); 2 Synergy Innovation Institute for Modern Industries, Guangdong University of Technology, Dongyuan 517500, China; zhfhu@gdut.edu.cn; 3 School of Physics Science and Technology, Lingnan Normal University, Zhanjiang 524048, China; xiewei@lingnan.edu.cn

**Keywords:** oxygen vacancies, WO_3−_*_x_* nanosheets, photocatalysis, plasmon resonance absorption, photodegradation

## Abstract

Oxygen vacancy defects play an important role in improving the light-capturing and photocatalytic activity of tungsten trioxide (WO_3_). However, the hydrogen treatment method that is commonly used to introduce oxygen vacancies is expensive and dangerous. Therefore, the introduction and control of oxygen vacancy defects in WO_3_ remains a challenge. Here, we demonstrated that oxygen vacancies could be successfully introduced into WO_3−*x*_ while using a facile method through low temperature annealing in alcohol. The obtained WO_3−*x*_ samples with optimal oxygen vacancies showed strong absorption of light, extending from the ultraviolet to the visible and near-infrared regions, and exhibits strong plasmon resonance from 400–1200 nm peaking at approximately 800 nm. When compared to pristine WO_3_, the photocatalytic activity of WO_3−*x*_ was greatly improved in the ultraviolet and visible regions. This study provides a simple and efficient method to generate oxygen vacancies in WO_3_ for photocatalysis, which may be applied in the photoelectrochemical, electrochromic, and photochromic fields. Because oxygen vacancy is a common characteristic of metal oxides, the findings that are presented herein may be extended to other metal oxides.

## 1. Introduction

Semiconductor photocatalysis has attracted significant attention due to its promising applications in solar energy conversion, since the discovery of water splitting on a titanium dioxide (TiO_2_) photoanode in the 1970s [1]. However, the photocatalytic efficiency of TiO_2_ is limited by its large band gap energy and fast electron-hole recombination due to its high density of trap states [2,3]. Great efforts have been dedicated to enhancing the visible light absorption of large band gap metal oxides. For instance, dye-sensitized and noble metal nanodot decorated TiO_2_ nanostructures were developed [4,5], forming heterojunctions with other semiconductors [6,7], and band gap narrowing was achieved via elemental doping in order to improve the conversion efficiency of metal oxide photoelectrodes. These methods modified the optical absorption coefficient and wavelength of the materials [8,9,10]. In addition to studies on reducing the band gap of TiO_2_ for enhanced visible light response, alternative semiconductor materials (WO_3_, ZnO, SnO_2_, ZnGaO_4_, and BiVO_4_) with intrinsic narrow band gaps are currently being explored [11,12,13,14]. Among them, tungsten trioxide is an attractive semiconductor material.

Tungsten trioxide (WO_3_) exhibits powerful oxidation properties, non-toxicity, and low cost and it has found widespread applications in electrochromic devices [15], gas sensors [16,17], photoelectrochemical water splitting, and photodegradation of organic compounds [18,19,20,21,22]. However, the photocatalytic activity of pristine WO_3_ is not high enough for practical use. Therefore, different strategies have been developed to increase the photocatalytic activity of WO_3_, including the preparation of WO_3_ with different nanostructures [23], supporting noble metal Pt/Au/Ag [24,25,26], composites with other materials [27,28,29], and oxygen vacancy defects [30,31,32,33,34]. The introduction of oxygen vacancies is a simple and efficient strategy to improve the photocatalytic performance of WO_3_ and it can greatly increase its conductivity and the absorption of visible light by local surface plasmon resonance (LSPR) in the near-infrared region, yielding an intensity that is comparable to bandgap absorption [35,36]. Over the past decades, persistent efforts have focused on improving the photocatalytic activity of WO_3_ by introducing oxygen vacancies, and various strategies have been proposed. At present, the main method for introducing oxygen vacancies is through the hydrogenation of WO_3_ [31,37]. Chen et al. produced black TiO_2_ using hydrogen treatment of white TiO_2_ in 2011, which triggered a surge in the production of hydrogenated semiconductors [38] and vacuum annealing procedures [37]. For instance, Yamashita et al. reported a facile H-spillover route to prepare heavily hydrogen-doped WO_3_ (H*_x_*WO_3_) [39]. These hydrogenated H*_x_*WO_3_ materials exhibited strong plasmonic absorption in the visible light region, which is tunable over a wide range by varying its stoichiometry. The electron concentration of the hydrogenated sample was high, up to 3.1 × 10^21^ cm^−3^. Gong et al. explored the exfoliation of layered tungstic acid to form WO_3_ nanosheets, and prepared sub-stoichiometric WO_3_ single crystal nanosheets, and subsequently introduced oxygen vacancies [35]. Simultaneously, the introduction of oxygen vacancies can promote the separation of photo-generated electrons and holes and inhibit their recombination, thus improving photocatalytic activity [40]. Therefore, the introduction of oxygen vacancies in WO_3_ plays an important role in photocatalytic performance. However, these methods require harsh experimental conditions and are expensive, which make them difficult to perform in most laboratories. Consequently, WO_3_-based semiconductors with adequate activity under visible or solar light are still under investigation. 

Herein, we prepared monoclinic structured WO_3_ nanosheets via a one-step template-free hydrothermal route and then introduced oxygen vacancies through low temperature annealing in alcohol. These processes successfully synthesized non-stoichiometric monoclinic structured WO_3_ (WO_3−*x*_) nanosheets. Because of the introduction of oxygen vacancies, the WO_3−*x*_ showed enhanced electron concentrations of up to 9.1 × 10^21^ cm^−3^, which are enough to induce LSPR. The WO_3−*x*_ also exhibited very strong visible and infrared light absorption, which significantly increased its photocatalytic activity.

## 2. Experimental

### 2.1. Preparation of the WO_3_ Nanosheets

The WO_3_ single crystal nanosheets were synthesized by a one-step template-free hydrothermal route [41]. Sodium tungstate dehydrate (Na_2_WO_4_·2H_2_O, 0.6398 g, 2 mmol) was added into distilled water (40 mL) and hydrochloric acid (40% HCl, 20 mL, adjusted pH = 1.5) was subsequently added into the Na_2_WO_4_ solution at room temperature (25 °C), stirring with vigorous magnetic for approximately 10 min. Then, the mixture was transferred to a 100 mL Teflon-lined stainless-steel autoclave. The autoclave was sealed and incubated at 180 °C for 12 h and then subsequently cooled down to room temperature naturally. The final products were collected by centrifuging the mixture, washed several times with distilled water and absolute ethanol, and drying at 60 °C in air. 

### 2.2. Preparation of WO_3−x_ Nanosheets

In a simple synthetic procedure, the prepared green-yellow WO_3_ nanosheets (0.5 g) were added into 10 mL absolute ethanol to form a suspension. After vigorous stirring for 10 min, the suspension was poured into a sintering boat (length 6 cm, width 3 cm, height 1.5 cm) and transferred to a vacuum tube furnace (SK-G06163, Φ60/50 × 1000 mm). The ends of the vacuum tube are sealed. The initial temperature is set to 50 °C, and the temperature is raised at a rate of 5 °C/min until the temperature reaches 400 °C, and the temperature is maintained at 400 °C for three hours. Then, the temperature is lowered at a rate of 5 °C/min until the temperature reaches 50 °C, and hence, cooled down to room temperature naturally. The obtained grey powders were collected for further characterization.

### 2.3. Characterization

The X-ray diffraction (XRD) patterns were recorded by X-ray diffractometer (D8 ADVANCE, BRUKER) with Cu-K_α_ radiation (λ = 1.541 Å) in a wide angle range from 10° to 80° on 2θ scale. Field emission scanning electron microscopy (FE-SEM) images, which were collected on a Hitachi SU8220 electron microscope with a working distance of 8 mm. Transmission electron microscopy (TEM) images and high-resolution TEM images were performed by using a FEI TecnaiG2 F20 with an acceleration voltage of 200 kV and TEM sample specimens were prepared by briefly ultrasonicating the sample powders in ethanol, followed by placing a drop of the suspension onto lacey support films that were dried before imaging. Fourier transformed infra-red (FT-IR) measurements were carried out using a 80/80 v Bruker TENSOR27 spectrometer. The FT-IR spectra were acquired with KBr discs under transmission mode. The scanned wavenumber range was from 4000 to 400 cm^−1^. The Raman spectra were recorded on a RENISHAW A-9570-2000 Raman spectrometer with an excitation wavelength of 785 nm. UV-Vis diffuse reflectance spectroscopy (DRS) measurement was conducted by a SHIMADZU UV-3600 spectrophotometer. X-ray photoelectron spectra (XPS) were recorded on a THERMO ESCALAB 250 Xi with a monochromatic Al K_α_ X-ray source. The electron paramagnetic resonance spectra were recorded while using a JES-FA200 spectrometer at room temperature. 

### 2.4. Photocatalytic Test

#### 2.4.1. UV Light Photocatalytic Degradation 

Rhodamine B (RhB) is an organic dye with a bright red colour and it is widely used as a model pollutant in photocatalytic tests. Therefore, the photocatalytic activity of the WO_3−_*_x_* nanosheets samples was evaluated in terms of the decolorization of RhB dye under UV irradiation from a 500 W Hg lamp. The lamp was positioned in a cylindrical Pyrex vessel and cooled by circulating water to maintain the reaction temperature at approximately 27 °C. A quartz tube was used as the photo catalytic reactor. The catalysts (0.02 g) were dispersed into 40 mL of 2 × 10^–5^ M RhB solution and stirred in the dark for 20 min to reach a complete adsorption–desorption equilibrium between the photocatalyst and RhB solution. Then, the mixture was exposed to UV light. Vigorous magnetic stirring was maintained to keep WO_3−*x*_ nanosheets suspended in the RhB solution. The concentration of aqueous RhB was determined with a UV-Vis spectrophotometer by measuring the absorption peak intensity at 553 nm.

#### 2.4.2. Visible Light Photocatalytic Degradation 

The photocatalytic activity of WO_3−*x*_ nanosheets under visible light was also evaluated by monitoring the decomposition of RhB. The apparatus for studying the photocatalytic decomposition of RhB was identical under visible and ultraviolet light, except that an 800 W Xe lamp with a 420 nm cut-on filter was used instead of a Hg lamp as the light source. 

## 3. Results and Discussion

### 3.1. Characterization of the WO_3−x_ Nanosheets 

**Figure 1:**
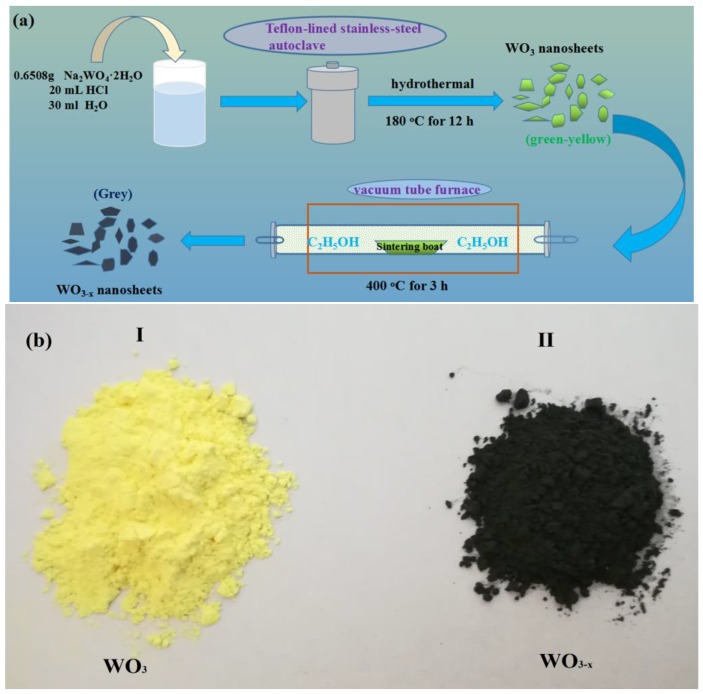
(**a**) Schematic illustration of the fabrication of tungsten trioxide (WO_3_) and non-stoichiometric monoclinic structured WO_3_ (WO_3−*x*_) nanosheets. (**b**) Digital photos of the WO_3_ (I) and WO_3−*x*_ (II) nanosheets samples.

The WO_3−*x*_ single crystal nanosheets were prepared by a one-step template-free hydrothermal route and oxygen vacancies were introduced through low-temperature annealing in ethanol. It is well-known that ethanol has reducing properties. When annealing WO_3_ in ethanol (anaerobic environment), ethanol can consume oxygen atoms in WO_3_, resulting in oxygen vacancies in the crystals. This strategy is simple, safe, and efficient when compared to the hydrogen treatment of WO_3_. The fabrication procedure of stable WO_3−*x*_ nanosheets is schematically illustrated in Figure 1a. Meanwhile, the annealing process was accompanied by a strong colour change from the original light yellow WO_3_ to the final grey WO_3−*x*_ (Figure 1b). This method can be used in most laboratories and mass production of WO_3−*x*_ can also be achieved. The obtained WO_3−*x*_ exhibits very good stability, as no change was observed after storage for six months. We performed detailed characterizations and photocatalytic performance tests of the prepared catalyst, as follows.

**Figure 2:**
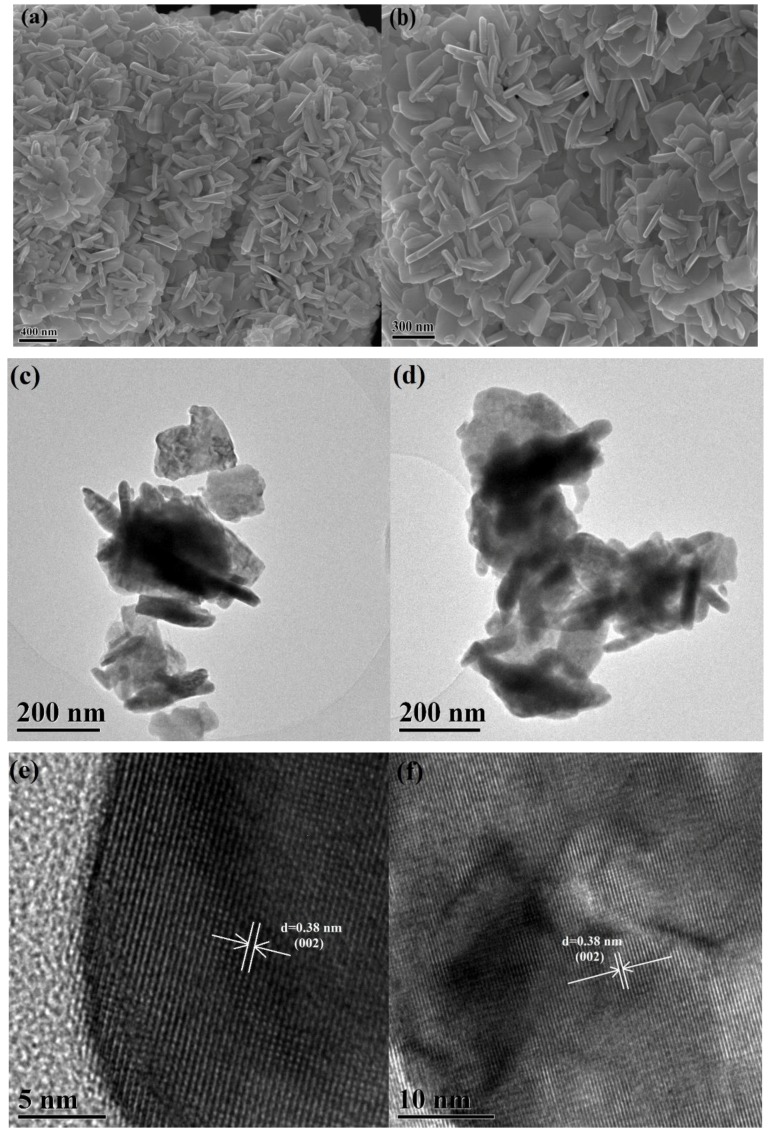
Field emission scanning electron microscopy (FE-SEM) images of the WO_3_ (**a**) and WO_3−*x*_ (**b**) nanosheets. FE-TEM images of the WO_3_ (**c**) and WO_3−*x*_ (**d**) nanosheets. Field emission transmission electron microscopy (HR-TEM) images of the WO_3_ (**e**) and WO_3−*x*_ (**f**) nanosheets.

The morphologies of the WO_3_ and WO_3−*x*_ samples were examined by FE-SEM and field emission transmission electron microscopy (FE-TEM), as shown in Figure 2. The SEM and TEM images clearly show the stacking morphology of the WO_3_ nanosheets and no obvious change in the size and morphology was observed after annealing. The thickness of the nanosheets was typically 20–30 nm, with a length of 100–150 nm. In addition, the similar morphologies of WO_3_ (Figure 2a,c) and WO_3−*x*_ (Figure 2b,d) prove that the annealing treatment at 400 °C does not significantly change the nanosheet morphology of WO_3−x_. As shown in the representative HR-TEM image of an unannealed WO_3_ nanosheets (Figure 2e), clear lattice fringes can be identified, and the lattice fringe spacing between two adjacent crystal planes is 0.38 nm, corresponding to the (002) lattice plane of the monoclinic structured WO_3_ [42]. In contrast, the framework of the highly crystalline WO_3−_*_x_* can be clearly observed in Figure 2f, and the WO_3−_*_x_* nanosheets also crystallize well and have a lattice spacing of 0.38 nm between the (002) planes. 

**Figure 3:**
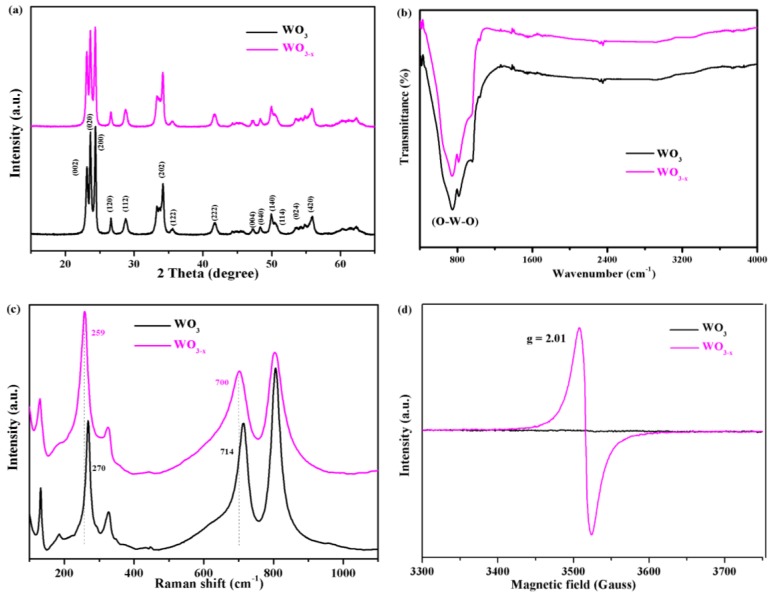
Comparison of the WO_3_ and WO_3−*x*_ nanosheets. (**a**) X-ray diffraction (XRD) patterns; (**b**) Fourier-transform infrared spectroscopy (FTIR) spectra; (**c**) Raman spectra and (**d**) electron paramagnetic resonance (EPR) spectra at room temperature.

The monoclinic crystal structure of the as-prepared WO_3−*x*_ and WO_3_ samples were confirmed by the X-ray diffraction patterns shown in Figure 3a. The WO_3_ and WO_3−*x*_ samples exhibited similar diffraction peaks, with peak centers at 23.1, 23.6, 24.3, 26.6, 28.7, 33.4, 34.1, 41.6, 50.1, and 55.9 corresponding to the (002), (020), (200), (120), (112), (202), (122), (222), (140), and (420) crystal faces of the monoclinic structured WO_3_ that was indexed to the JCPDS card No. 43-1035 [43]. No characteristic peaks of other crystalline impurities were detected. In addition, no other crystalline forms of WO_3_ were observed after low temperature annealing.

To further study the chemical structure and surface composition of the as-fabricated WO_3−*x*_ nanosheets, the samples were characterized, as shown in Figure 3b. Both samples only show a strong absorption peak at 800 cm^−1^, which can be attributed to (O–W–O) of the interconnected octahedron WO_3_ [44]. Meanwhile, no other characteristic peaks were observed after low temperature annealing. This indicates that no other chemicals or organic residues were present on the surface of the WO_3−*x*_ nanosheets after the fabrication procedure. Raman spectroscopy was performed to investigate the chemical structure of the samples, as shown in Figure 3c. The oxygen deficiencies of WO_3−*x*_ were unambiguously supported by the raman spectroscopy data. The raman spectra of the WO_3_ nanosheets display three major characteristic peaks at 270, 715, and 805 cm^−1^, arising from the bending vibration of δ_(O–W–O)_ and stretching vibrations of ν_(W–O–W)_ of the monoclinic phase, respectively [35]. However, the characteristic raman peaks for WO_3−*x*_ broaden after annealing, thus suggesting slight changes in its structure. After annealing, the W^6+^–O stretching band at 270 and 715 cm^−1^ slightly shifted to lower wavelengths of 258 and 700 cm^−1^. Similar results were reported in previous studies [35]. These results indicate that oxygen vacancies were successfully introduced to the nanosheets. The formation of oxygen vacancies in the WO_3_ crystals caused an up-shift of the W^6+^-O bond in the raman spectrum, indicating that the structure of WO_3−*x*_ was changed by the formation of oxygen vacancies. These results are consistent with the HR-TEM images of WO_3−*x*_ (Figure 2d).

To further confirm the presence of oxygen vacancies in WO_3−*x*_, electron paramagnetic resonance (EPR) spectroscopy was performed. EPR is highly sensitive to paramagnetic species containing unpaired electrons and it has been widely used to characterize oxygen vacancies. The test was performed at room temperature and the analytical results are shown in Figure 3d. It is evident that the WO_3_ nanosheets did not contain any paramagnetic sites, as a flat line was observed in its EPR spectrum. In contrast, a strong EPR signal was observed at *g* = 2.01 and it could be ascribed to trapped electrons on oxygen vacancies in the WO_3−*x*_ [34,45].

**Figure 4:**
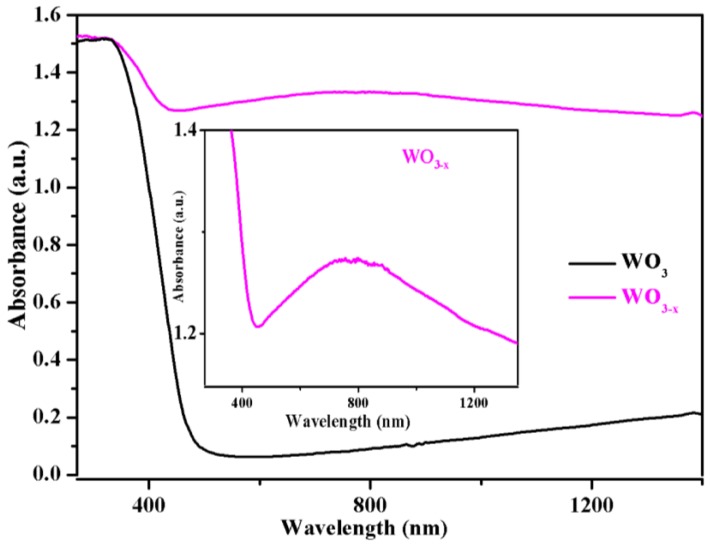
UV-Vis diffuse reflectance spectra of the WO_3_ and WO_3−*x*_ nanosheets.

The optical properties of the pristine WO_3_ and WO_3−*x*_ nanosheets were examined by measuring their UV-Vis diffuse reflectance spectra, as shown in Figure 4. For the two samples, the steep increase in absorption at wavelengths shorter than ≈ 476 nm (2.6 eV) can be attributed to the intrinsic bandgap absorption of crystalline pristine WO_3_. When examining the optical absorption beyond the band edge, WO_3−*x*_ possess a significant absorption in the visible and near infrared region. Notably, there is a broad absorption band from 400 to 1200 nm peaking at approximately 800 nm (see the inset of Figure 4). The absorption spectrum is similar to the reported results for WO_2.83_ nanorods by Alivisatos et al. [46] and Meso-WO_2.83_ by Thomas et al [45]. The enhanced visible light absorption peak in WO_3−*x*_ nanosheets samples can be undoubtedly assigned to the LSPR. The surface plasmon resonance in WO_3−*x*_ is correlated with its abundant free electrons. It is well known that even a very small decrease in oxygen content yields a dramatic increase in the electrical conductivity of WO_3_. Consequently, oxygen vacancies provide abundant localized electrons in WO_3−*x*_ to support its plasmon resonance, in contrast to the pristine WO_3_. Thus, we further estimated the free-carrier density from the LSPR peak, according to the Drude model [45,46].

At the resonance condition, the plasmonic frequency, ω_sp_ can be expressed as:

(1) $$\omega_{sp}=\sqrt{\frac{\omega_{p}{}^{2}}{1+2\varepsilon_{m}}-\gamma^{2}},$$

where ω_p_ is the bulk plasma, ε_m_ is the dielectric constant of the surrounding medium, and *γ* is the damping parameter that is equal to the line width of the plasmon resonance band [45]. In our case, the value of ε_m_ is 1. For WO_3−*x*_, the resonance energy is 1.55 eV at the plasmonic wavelength (800 nm) and the line width is 0.66 eV, as obtained by measuring the full width at half-maximum (FWHM) of the optical spectrum. Therefore, the bulk plasma frequency, ω_p_, was estimated to be approximately 2.92 eV. Moreover, ω_p_ depends on the free electron density, N, by the formula:

(2) $$\omega_{P}=\frac{{\mathrm{Ne}}^{2}}{\varepsilon_{0}m*},$$

where e is the elementary charge, *ε*_0_ is the permittivity of free space, and m* is the effective mass of the free carriers. According to a previous report [46], the effective mass of WO_3−*x*_ is in the range of m_0_ to 1.4 m_0_. Therefore, the effective electron mass of WO_3−*x*_ was set to m* = 1.2 m_0_, and m_0_ represents the electron rest mass. Therefore, the free electron concentration, N, was estimated to be 9.1 × 10^21^ cm^−3^ in the WO_3−*x*_ sample. Table 1. summarizes the free electron concentration of a previously reported plasma-doped WO_3−*x*_.

**Figure 5:**
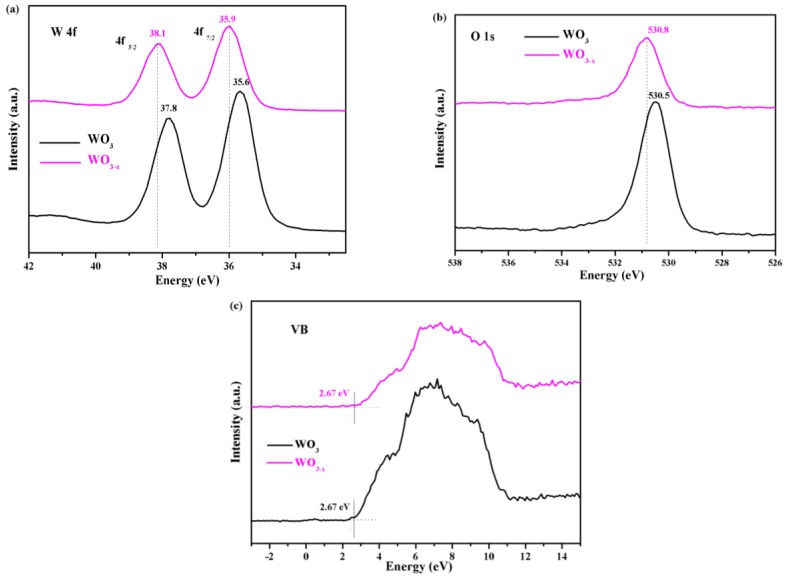
(**a**) The Ti 2p X-ray photoelectron spectra (XPS) spectra of the as-prepared WO_3_ and WO_3−*x*_; (**b**) O 1s XPS spectra of the as-prepared WO_3_ and WO_3−*x*_ and (**c**) valence band (VB) XPS spectra of the as-prepared WO_3_ and WO_3−*x*_.

X-ray photoelectron spectroscopy (XPS) was employed in order to investigate the transformation of surface chemical bonds and detect the electronic valence band position of the samples. The W 4f_7/2_ and 4f_5/2_ XPS peaks of WO_3_ are centred at binding energies of 35.6 and 37.8 eV, respectively, as shown in Figure 5a, which is typical for W^6+^–O bonds in WO_3_ [35]. For WO_3−*x*_, the W 4f XPS spectrum shows different features, where the W 4f_7/2_ and 4f_5/2_ peaks shift to higher energies at 35.9 and 38.1 eV, respectively (a shift of 0.3 eV). The XPS spectra of O 1s (Figure 5b) in the WO_3_ samples show a well-developed peak at 530.4 eV, which can be attributed to the lattice oxygen. However, the O 1s XPS peak of WO_3−*x*_ (Figure 5b), which is located at ~530.7 eV, also showed a clear positive shift in binding energy of approximately 0.3 eV, which is consistent with shift that was observed in the W 4f peak. A reasonable explanation can be given for the shifting phenomena. Upon removal of an O atom, the nearest W atoms relax away from the vacancy, strengthening their bonding with the rest of the lattice. This outward relaxation decreases the overlap between the W dangling bonds and reduces the W–O bond length. To maintain the original crystal structure and stability, the W^6+^–O bond binding energy was increased [47,48,49]. Reasonably, the positive shifts in the binding energies of W 4f and O 1s can be ascribed to the strong interaction between W^6+^ and oxygen vacancies. Van de Walle et al*.* showed that when the oxygen atoms are removed, the outward motion of the W atoms cause their bonds with the next-nearest-neighbour O atoms along the W–Vo–W direction to be compressed by up to 20%, through hybrid density functional theory [47]. Therefore, the positive shift of the XPS peak can be attributed to a shortening of the correlation length because of the presence of oxygen vacancies. Similar results have been reported for TiO_2−*x*_ by Dal Santo et al. [50]. They observed a lattice contraction of TiO_2_ induced by the presence of oxygen vacancies.

The valence band (VB) position was analysed through the VB XPS spectra of the samples (Figure 5c). Both VB spectra were similar and the VB maxima were estimated by linear extrapolation of the peaks to the baselines, which derives a band edge position of 2.76 eV below the Fermi energy for both samples. The annealing treatment exerted a negligible effect on the VB position of the WO_3−*x*_ surface.

### 3.2. Photocatalytic Degradation Test

**Figure 6:**
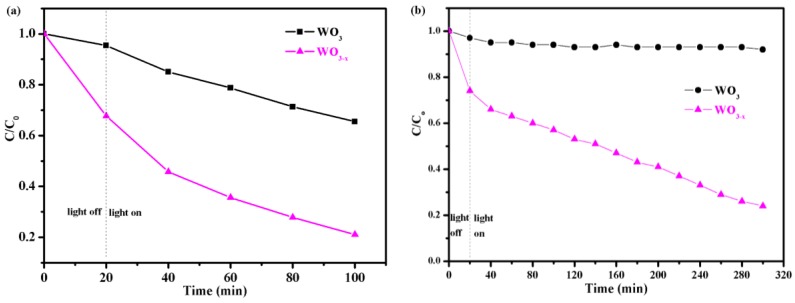
(**a**) UV light photocatalytic degradation of rhodamine B (RhB) and (**b**) visible-light photocatalytic degradation of RhB by the prepared photocatalysts.

To evaluate the ability of the catalysts to degrade organic contaminants, RhB was used as a representative pollutant. The as-prepared WO_3−*x*_ photocatalysts are expected to exhibit considerable UV-light photocatalysis as compared to pristine WO_3_. Before irradiation, an adsorption experiment was performed in the dark to ensure that adsorption equilibrium of RhB on the catalyst surface was adequately established. The results are shown in Figure 6a, where t, C, and C_0_ refer to the irradiation time, instantaneous RhB concentration, and RhB concentration before irradiation, respectively. The C/C_0_ ratio was used to describe the decomposition efficiency, which represents the concentration ratio before and after a certain period of reaction time. The WO_3−*x*_ sample efficiently decomposed the RhB in 80 min under in the irradiation of UV light, while the pristine WO_3_ decomposed only approximately 30% of the RhB. The annealing-treated WO_3−*x*_ samples exhibited better photocatalytic activity than the pristine WO_3_. 

**T**he photocatalytic activity of the WO_3−*x*_ sample was also investigated under visible light (λ > 420 nm) illumination. As shown in Figure 6b, the WO_3_ samples showed little ability to decompose RhB after visible light irradiation for 320 min. In contrast, the WO_3−*x*_ samples exhibited higher photocatalytic efficiency (77%) than that of WO_3_ (9%). The photocatalytic activity of visible light was greatly improved, because WO_3−*x*_ has enhanced visible light absorption at 400–1400 nm and the plasmonic effect due to the introduction of oxygen vacancies (Figure 3a). The oxygen vacancies were demonstrated to be electron donors and contributed to the enhanced donor density [44]. The increased donor density improved charge transport in WO_3−*x*_. Therefore, the LSPR and charge transport are believed to be major mechanisms for the observed photocatalytic activity enhancement in the WO_3−*x*_ samples.

## 4. Conclusions

We prepared monoclinic structured WO_3_ nanosheets via a one-step template-free hydrothermal route and then introduced oxygen vacancies through low temperature annealing in alcohol. _−*x*_ possesses a high concentration of oxygen vacancies, which were demonstrated to be electron donors and contributed to the enhanced donor density, which is believed to induce LSPR and to increase light harvesting efficiency in the visible and infrared regions. Simultaneously, the oxygen vacancies act as traps for reducing the recombination of electrons and holes and significantly improved the e-h separation efficiency, greatly enhancing the photocatalytic activity. This represents a novel approach for further improving the photocatalytic properties of WO_3_. In addition, this study illustrates a facile route to obtain plasmonic WO_3−*x*_ nanosheets and may offer a generalized method for generating other semiconductor nanostructures with LSPR, such as deficient vanadium and molybdenum oxides.

## Figures and Tables

**Table 1:** The free electron concentration of a previously reported plasma-doped WO_3−*x*_.

Plasmonic Materials	LSPR Wavelength	Free-Carrier Density (cm^−^^3^)	Reference
WO_3−_*_x_*	800 nm	9.1 × 10^21^	This Work
WO_2.83_	650 nm	9.79 × 10^21^	Ref. [45]
WO_3−_*_x_*	1450 nm	2.5 × 10^21^	Ref. [35]
WO_3−_*_x_*	900 nm	6.3 × 10^21^	Ref. [46]
